# Improvement of Moist Heat Resistance of Ascorbic Acid through Encapsulation in Egg Yolk–Chitosan Composite: Application for Production of Highly Nutritious Shrimp Feed Pellets

**DOI:** 10.3390/ani12182384

**Published:** 2022-09-13

**Authors:** Jidapa Jaroensaensuai, Saowakon Wongsasulak, Tipaporn Yoovidhya, Sakamon Devahastin, Wanilada Rungrassamee

**Affiliations:** 1 Department of Food Engineering, Faculty of Engineering, King Mongkut’s University of Technology Thonburi, Tungkru, Bangkok 10140, Thailand; 2 Food Technology and Engineering Lab, Pilot Plant Development and Training Institute, King Mongkut’s University of Technology Thonburi, Tha-Kham, Bang Khun Thian, Bangkok 10150, Thailand; 3 The Academy of Science, The Royal Society of Thailand, Dusit, Bangkok 10300, Thailand; 4 Microarray Research Team, National Center for Genetic Engineering and Biotechnology, 113 Thailand Science Park, Phahonyothin Road, Khlong Nueng, Khlong Luang, Pathum Thani 12120, Thailand

**Keywords:** ascorbic acid, chitosan, egg yolk, feed pellets, microencapsulation, shrimp

## Abstract

**Simple Summary:**

Egg yolk (EY) is an excellent supplement for aquatic animals and has good food functionality. According to the high lipid content in EY, it was, for the first time, used in combination with chitosan (CS) to encapsulate the ascorbic acid (AA) to minimize the loss of AA during exposure to feed processing and seawater. The microcapsules’ production yield, EE, and moist heat resistance were evaluated. One selected encapsulated AA was fortified in shrimp feed. The AA retention in feed processing and seawater was evaluated. Both EE and production yields of the microcapsules were relatively high compared to other reports. Moist heat resistance capability of the encapsulated AA was up to 82%. EY was essential in moist heat protection, while CS significantly improved the microcapsules’ production yield, EE, and morphology. The loss of AA in feed processing and seawater was remarkably improved by 16 folds compared to the unencapsulated AA. The microcapsules showed high potential application for foods and aquatic feed to protect heat-labile and hydro-soluble substances.

**Abstract:**

Egg yolk (EY) is an excellent supplement for aquatic animals and has good technofunctionality. Ascorbic acid (AA) is a potent bioactive substance and is essentially added to shrimp feed; however, it is drastically lost in both feed processing and in rearing environments. In this study, AA was microencapsulated in an EY–chitosan (CS) composite. The encapsulated vitamin was then mixed into a shrimp feed mixture to form pelleted feed via twin-screw extrusion. The effects of the EY/AA ratio and the amount of CS on moist heat resistance, production yield, encapsulation efficiency (EE), and morphology of microcapsules were investigated. The molecular interaction of the microcapsule components was analyzed by FTIR. The size and size distribution of the microcapsules were determined using a laser diffraction analyzer. The microstructure was evaluated by SEM. The physical properties of the microcapsule-fortified pelleted feed were determined. The AA retention at each step of feed processing and during exposure to seawater was evaluated. The results showed that the microcapsules had a spherical shape with an average diameter of ~6.0 μm. Decreasing the EY/AA ratio significantly improved the production yield, EE, and morphology of the microcapsules. EY proved to be the key component for moist heat resistance, while CS majorly improved the production yield, EE, and morphology of the microcapsules. The microcapsules showed no adverse impact on feed properties. The loss of AA in food processing and seawater was remarkably improved. The final content of the encapsulated AA remaining in shrimp feed was 16-fold higher than that of the unencapsulated AA.

## 1. Introduction

Shrimp farming is a quickly growing global industries driven by the continuously increasing global demand for crustaceans [1,2]. It is projected that shrimp production will need to increase by up to 50% by 2050 to meet global demand [2]. To maximize shrimp production yield, higher feed quality and better aquaculture technologies are needed. To achieve optimal production yield, various nutrients and bioactive compounds have been added to the feed, such as vitamins, minerals, albumin, lecithin, phospholipids, fish oils, astaxanthin, prebiotics, and probiotics to improve shrimp growth performance and survival rate [3,4]. Among feed supplements, ascorbic acid (AA) is one of the essential nutrients needed for shrimp nutrition. It plays a vital role in aquatic animal health as a free-radical scavenger and cofactor in a number of biochemical reactions [5,6,7]. Ascorbic acid deficiency in shrimp can result in poor growth, reduced molting frequency (or even incomplete molting), low stress resistance, impaired collagen synthesis, and delayed wound healing [8]. Unfortunately, AA is extremely sensitive to oxygen, light, and heat, especially the inevitable moist heat used in the shrimp feed production process. It is also water-soluble [9,10]. The loss of AA fortified in aquaculture feed is up to 80% during processing, storage, and in seawater [9,11]. Over the past two decades, many efforts have been made to encapsulate drugs, vitamins, enzymes, and vaccines to maintain the efficacy of bioactives and maximize the biofunctionality of the aquafeeds [9,11,12]. Nonetheless, some serious concerns on the practicability of the encapsulation remain, for instance, toxicity, biocompatibility, and bioavailability of the encapsulation materials. Moreover, sufficient resistibility of the encapsulation system to the harsh conditions of feed processing remains critical and highly challenging [11,12,13]. The presence of lipids in the encapsulation-wall matrix effectively reduces the degradation/loss of heat-sensitive compounds. Recently, we successfully developed moist heat-resistant microcapsules to maximize the viability of encapsulated probiotics exposed to the steaming process [14]. Solid lipids added to the encapsulation matrix have been reported to reduce the migration of thermal water vapors into the microcapsules, thereby significantly increasing the encapsulated probiotic survivability [15]. The encapsulated AA in alginate and gum arabic shows better stability up to 188 °C. Indeed, in combination with moist heat, shear/friction force and pressure generating in the extrusion and pelletizing processes cause exponential damage to vitamins. The thermal resistibility of the encapsulated AA should thus be evaluated in actual feed processing rather than simulated conditions. Nonetheless, research conducted on true feed processing is limited [13,16].

As mentioned, the presence of lipids in the encapsulation-wall matrix significantly helps improve thermal stability for the core compound. Fresh hen egg yolk (EY) is mainly composed of 32% lipids and 16% proteins with a moisture content of about 50–52%. The structural composition of EY is in the form of granules suspended in the plasma fraction, with a composite ratio of 22 to 78. The major component of the granule fraction is proteins, while the main fraction of plasma is lipids. The plasma lipids account for 97% of the total lipids in EY [17]. The main lipid form in the plasma is low-density lipoprotein (LDL), which are in the form of core lipids surrounded by a monofilm of phospholipids and proteins [18,19]. LDL plays a critical role in emulsification and interfacial properties between oil and water [18,19]. EY LDL has also been used as an encapsulation material to enhance the bioavailability of micronutrients [20]. EY lipids importantly promote shrimp growth, improve lipid utilization, and reduce shrimp mortality [21,22].

Due to the structural composition, functionality, and biological activities of EY, its potential use as an encapsulation material for aquafeed application would be interesting. We hence conducted a preliminary study to encapsulate AA with fresh hen EY via spray-drying. It was found that the spray-drying yield was only 30%, which was slightly lower than the common range of encapsulation yield. In addition, the microcapsules were sticky. The other undesirable properties of the spray-dried lipid-rich products are poor-drying yield and morphology, sticky surface, and high lipid oxidation [23].

The problems could be overcome by combining the lipids with other biopolymers. Chitosan is one of the most used materials to form composites with lipids due to its property to be protonated in an acidic environment and interact with the negatively charged groups of the other biopolymers. The use of chitosan–lipid complexes as an encapsulation material and delivery vehicle has been reported [24,25,26]. It has been found that CS-EY protein composite improves the stability of the encapsulated bioactive compounds [19]. In addition, chitosan is noted as an immunostimulant for aquatic animals [27,28].

This work was conducted to investigate the effectiveness of fresh hen EY as an encapsulation material for a hydrosoluble and thermal-sensitive vitamin during exposure to feed processing and seawater. CS was used as a coencapsulation material with EY, and AA was selected as the model vitamin. The stability of the encapsulated AA fortified in shrimp feed at each step of feed processing and after 6 h exposure to seawater was investigated.

## 2. Materials and Methods

### 2.1. Materials

Fresh hen eggs (Betagro, Lopburi, Thailand) were purchased from a local supermarket. The chemical composition of the egg consists of approximately 75% moisture content, 14% proteins, 10% lipids, and 1% carbohydrates L-ascorbic acid was purchased from Thai Food and Chemical Co., Ltd. (Bangkok, Thailand). Chitosan (CS) flakes from shrimp shells with a molecular weight of 350 kDa and a degree of deacetylation (DD) of 93% (Chito Clear, TM4737) were purchased from Primex (EHF, Siglufjörour, Iceland). Glacial acetic acid (99.85%), sodium hydroxide (NaOH), hydrochloric acid (HCl), and sodium chloride (NaCl) were purchased from Carlo-Erba (AR grade, Val-de-Reuil, France). Commercial shrimp feed powder was obtained from Thai Union Feedmill Public Co., Ltd. (Samut Sakhon, Thailand).

### 2.2. Microencapsulation of AA in EY-CS Composites

The ascorbic acid solution was prepared by dissolving L-ascorbic acid in HPLC-grade water using a magnetic stirrer (IKA, C-MAG HS7, Staufen, Germany). The solution was then homogenized with fresh EY by using a high-speed homogenizer (IKA, Model T25 digital Ultra-Turrax, Werke GmbH & Co. KG, Staufen, Germany) at 300 rpm for 15 min to obtain a homogeneous emulsion. The temperature of the sample during homogenization was controlled at 25 ± 1 °C. Sodium hydroxide (1.0 N) and HCl (1.0 N) were used to adjust the pH of the homogenized solution to 7.50 ± 0.1. A stock solution of CS with a concentration of 2.5% (*w*/*v*) was prepared by dissolving CS in 1% (*w*/*v*) acetic acid with constant stirring at 300 rpm for 1 h using a magnetic stirrer (Model C-MAG HS7, IKA, Guangzhou, China). The CS solution was then added to the EY-AA emulsion and mixed at room temperature using a magnetic stirrer at 60 rpm for 15 min. The final solid content of the emulsion sample was set at 32.5% (*w*/*v*); mass concentration of CS in the emulsion was 1.5 and 6.0% of EY (*w*/*w*). The sample without CS was used as a control. The emulsion sample was placed in a spray dryer (Büchi, B-290, Flawil, Switzerland) to form microcapsules at an inlet temperature of 138 ± 2 °C and a feed rate of 4 mL/min; the air flow rate and nozzle pressure were maintained at 8 L/min and 0.4 bar, respectively. The spray-drying conditions were set based on our preliminary study, in which spherical microcapsules with minimal collapse structure and a desired moisture content of 12% wet basis (w.b.) were generated. The value of the moisture content was determined regarding the regulation of the Thai Industrial Standards Institute (TISI, 1993) for shrimp pellet mills that it should not exceed 12% (w.b.). Moreover, the highest production yield with acceptable encapsulation efficiency (~53%) was obtained under this spray-drying condition.

### 2.3. Determination of Production Yield

Determination of the production yield of the microcapsules was performed using the modified methods of Jain et al. (2015) [29]. The spray-dried microcapsules were collected and weighed. The production yield was calculated as per Equation (1).

(1) $$\mathrm{Production}\;\mathrm{yield}\;(\%)=\frac{M_{m}\times 100}{M_{i}}$$

where $M_{m}$ is the mass of the resulting microcapsules (g), $M_{i}$ is the total mass of CS, EY and AA taken to prepare the microencapsulation emulsion (g).

### 2.4. Determination of Encapsulation Efficiency

Encapsulation efficiency (EE) of microcapsules was determined based on the method of Jain et al. [29] with slight modification. Unencapsulated AA or excess AA deposited on the microcapsule surfaces was firstly removed by rinsing 0.02 g of the microcapsules with HPLC-grade water for 5 min; the capsules were then added to 50 mL of the solution of 0.1% (*w*/*v*) acetic acid and acetate buffer (20 mM, pH 4.0). The content was vigorously stirred until the microcapsules were completely dissolved (~20 min). The solution was filtered through a filter membrane (diameter of 0.45 µm). The AA content was then determined using high-performance liquid chromatography (HPLC). The HPLC (Water Alliance 2695) was equipped with a Waters 2998 photodiode array detector (Waters, Milford, MA, USA). Vertisep™ AQ8 C18, 5 μm (4.6 × 150 mm) column (Vertical Chromatography Co., Ltd., Nonthaburi, Thailand) was used in the analysis. The mobile phase was a degassed solution of 0.2 M KH_2_PO_4_ that was performed at a flow rate of 0.5 mL/min. Amount of detected AA was measured at a wavelength of 254 nm. Standard curve of AA with an R^2^ ≥ 0.99 was prepared by injecting AA standard into the extraction solvent at five concentrations (0, 100, 200, 300 and 400 μg/mL. EE was calculated using Equation (2).

(2) $$\mathrm{EE}\;(\%)=\frac{\mathrm{Encapsulated}\;\mathrm{AA}\;(g)\times 100}{\mathrm{Total}\;\mathrm{AA}\;\mathrm{taken}\;\mathrm{to}\;\mathrm{prepare}\;\mathrm{the}\;\mathrm{sample}\;(g)}$$

### 2.5. Determination of Moist Heat Resistance of Microcapsules

The microcapsules prepared as described in Section 2.2 were subject to steaming to study their moist heat resistance. Microcapsules were spread on a glass plate and steamed over a water bath (Major Science, SWB-10L-1, Saratoga, CA, USA). The steaming temperature was controlled at 95 ± 1 °C, while steaming time was 5 min. After the steaming, the capsules were removed from the water bath. The encapsulated AA was extracted by dissolving 20 mg of the microcapsules in 50 mL of mixed solution of 0.1% (*w*/*v*) acetic acid and acetate buffer (20 mM, pH 4.0). The content was vigorously stirred until the microcapsules were completely dissolved (~20 min). The solution was then filtered through a filter membrane (*D* = 0.45 µm). The amount of AA was determined by HPLC as per the methods outlined in Section 2.4. Moist heat resistance of the microcapsules was calculated using Equation (3).

(3) $$\mathrm{Moist}\;\mathrm{heat}\;\mathrm{resistance}\;(\%)=\frac{EA_{s}\times 100}{EA_{o}}$$

where $EA_{o}$ and $EA_{s}$ are the amounts of the encapsulated AA (g) before and after steaming, respectively.

### 2.6. Fourier-Transform Infrared Spectroscopy

Molecular structure characteristics of microcapsules and their changes were determined by Fourier-transform infrared (FTIR) spectroscopy. The microcapsules were prepared as per the method described in Section 2.3 and were introduced to FTIR spectrometer (Thermo Fisher Scientific, Nicolet 6700, Waltham, MA, USA). FTIR spectra were recorded from 3800 to 600 cm^−1^ at a resolution of 4.5 cm^−1^ by 32 scans. All samples were characterized in duplicate.

### 2.7. Determination of Microcapsules Morphology

The morphology of microcapsules was examined using a scanning electron microscope (JEOL, JSM-5410LV, Tokyo, Japan). Microcapsules were mounted on a stainless-steel stub and sputter-coated with gold at a coating condition of 10 kV, 5 mA for 2 min, with an Argon backfill of 10 Pa. Micrographs were recorded at a magnification of 1000× and 5000× and at a resolution of 1280 × 960 pixel.

### 2.8. Determination of Particle Size Distribution

Particle size distribution and median particle size (*D*_0.5_) of microcapsules were determined by laser diffraction using a Malvern Mastersizer 3000 analyzer (Malvern Instruments, Worcestershire, UK). Microcapsules were directly suspended in a dispersion system (Hydro EV) prior to the measurement.

### 2.9. Production of Pelleted Feed and Evaluation of Its Characteristics

Microcapsules produced at the optimal ratio of EY to AA with the optimal percentage of CS were fortified into shrimp feed to produce feed pellets via the process of extrusion. All pelleted feed samples were evaluated for their physical properties, encapsulation behavior and loss of AA.

#### 2.9.1. Production of Pelleted Feeds

Three types of pelleted shrimp feeds were produced: (1) plain pelleted feed (pelleted feed without AA), (2) pelleted feed containing unencapsulated AA and (3) pelleted feed containing encapsulated AA. The unencapsulated and the selected encapsulated AA were fortified in shrimp feed mixture at an AA ratio of 0.2% of feed (d.b.). The composition of the shrimp feed samples is shown in Table 1. All three pelleted samples were produced using the same formula and the same processing condition. Each feed was extruded through a 2 mm orifice die of a corotating twin-screw extruder (Hermann Berstorff, Model ZE25 × 33D, Hannover, Germany). The extruder was operated at a screw speed of 250 rpm, feeding speed of 35 rpm and cutting speed of 100 rpm. Extruded feed pellets were dried in an oven at 90 °C for 1 h to reach a final moisture content of 12% (w.b.), then cooled to room temperature of around 30 ± 1 °C. Pellets with a particle size of about 2 mm were obtained via sieving. The extrusion was employed for pelleted feed production according to the study of Yang et al. (2020) [13], which exhibited that the extrusion generally damaged the active ingredients fortified in the feed more than the pelletizing process. Extrusion was therefore chosen to produce pelleted feed samples that were subsequently evaluated for AA retention in the feed.

#### 2.9.2. Determination of Moisture Content and Bulk Density

Moisture content of the pelleted feed was defined as the mass of water in the pelleted feed compared to the dry mass of the feed. The value was gravimetrically determined by-drying 2 g of the feed in a hot air oven (Memmert, UF110, Schwabach, Germany) at 100 °C until a constant mass was obtained (AOAC, 1990). The moisture content is reported in dry basis. The moisture content was calculated as per Equation (4).

(4) $$\mathrm{Moisture}\;\mathrm{content}\;\mathrm{of}\;\mathrm{feed}\;(\%)=\frac{M_{w}}{M_{f}}\times 100$$

where $M_{w}$ is mass of the water in the pelleted feed (g) and $M_{f}$ is dray mass of the pelleted feed (g). Bulk density of pelleted feed was determined using the liquid replacement method; *n*-heptane (99%) was used as the working liquid.

#### 2.9.3. Determination of Sinking Velocity

Determination of the sinking velocity of pelleted feed in seawater was performed using the modified methods of Rout and Bandyopadhyah (1999) [30]. The determination was conducted by gravitationally dropping the feed into 1 L of seawater (3% salt, pH 8 and temperature of 25 °C) contained in an acrylic cylindrical pipe of 30 cm in height and 6.5 cm in diameter. A depth of 25 cm was set to determine the sinking velocity (in cm/s). Each reported datum is an average of ten measurements.

#### 2.9.4. Determination of AA Content

The amounts of AA contained in pelleted feed both before and after extrusion as well as after hot air-drying were determined by dissolving 2 g of ground feed sample in 50 mL of mixing solution of 0.1% (*w*/*v*) acetic acid and acetate buffer (20 mM, pH 4.0), then vigorously stirred using a vortex mixer until the sample were completely dissolved (~30 min). The solution was filtered through a filter membrane (*D* = 0.45 µm). The amount of AA was then determined by HPLC using the condition described in Section 2.4.

#### 2.9.5. Determination of Stability of Pelleted Feeds and Leaching of AA into Seawater

Physical stability of pelleted feed in seawater was determined by the horizontal shaking method of Obaldo et al. (2002) [31]. The shaking speed was set at 100 rpm, while the agitating speed was the one that initiated pellet motion at a stroke distance of 2 cm. The shaker’s tray held eight 250-mL flasks for each test run; each flask was filled with 100 mL of seawater and 2 g of feed pellets. The samples were periodically collected by removing them from seawater after immersion for 2, 4, 6 and 8 h. All remaining solids were collected using a Buchner filtration apparatus with Whatman filter paper no. 3 (pore size of 5 µm). Recovery solids and original feed samples were dried in an oven at 105 °C until a constant mass was obtained (~24 h). Stability of the pellets in seawater in terms of dry mass retention at time interval of incubation was calculated using Equation (5). The amount of AA remaining in the feed pellets was also measured according to the methods described in Section 2.5.

(5) $$\mathrm{Stability}\;\mathrm{in}\;\mathrm{seawater}\;(\%)=\frac{M_{t}\times 100}{M_{0}}$$

where $M_{0}$ and $M_{t}$ are the dry mass of pelleted feed before and after exposure to seawater (g), respectively.

### 2.10. Statistical Analysis

Experimental data were analyzed via one-way analysis of variance (ANOVA) and the results are reported as means with standard deviations. Tukey’s multiple comparison tests were used to establish significances between the mean values at a confidence level of 95%. All statistical calculations were performed using SPSS software version 17 (SPSS Inc., Chicago, IL, USA). All measurements were performed in triplicate, unless otherwise stated.

## 3. Results and Discussion

### 3.1. Effects of the Ratio of EY to AA on Production Yield, Encapsulation Efficiency, and Moist Heat Resistance of Microcapsules

**Figure 1:**
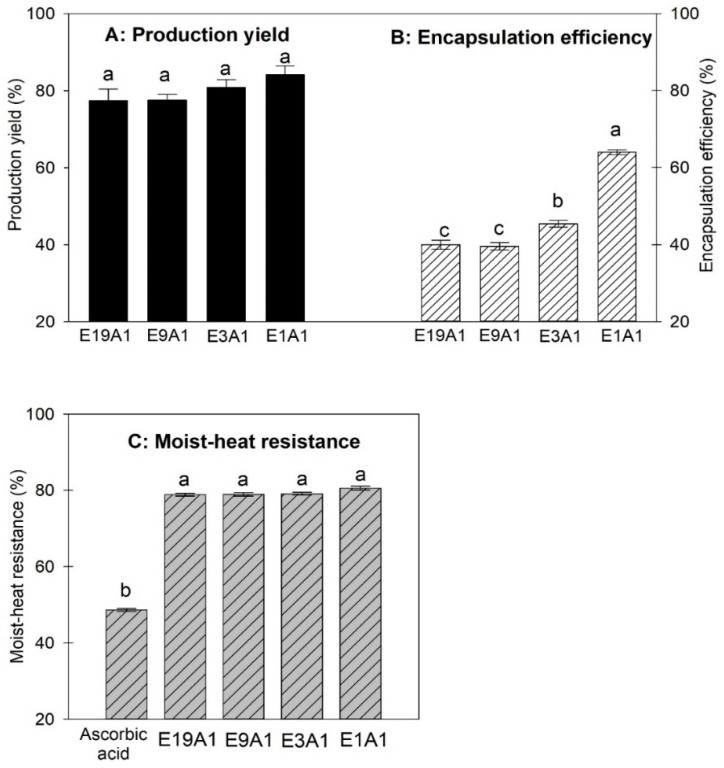
(**A**) Production yield of microcapsules prepared from different EY-AA mass ratio; E19A1, E9A1, E3A1 and E1A1. (**B**) Encapsulation efficiency of microcapsules prepared from different EY/AA mass ratio; E19A1, E9A1, E3A1 and E1A1. (**C**) Moist heat resistance of unencapsulated AA and microencapsulated AA E19A1, E9A1, E3A1 and E1A1.The different superscripts in each figure indicate significant differences of the values (*p* < 0.05).

Four microcapsules, E19A1, E9A1, E3A1 and E1A1, were prepared using an EY/AA mass ratio of 95/5, 90/10, 75/25 and 50/50, respectively. CS was used in combination with EY at a mass ratio of 1.5% of EY. Production yields of the four samples are shown in Figure 1A. The production yield increased as the ratio of EY/AA decreased. E1A1 exhibited the highest yield of ~84%. E3A1, E9A1, and E19A1, which were the microcapsules formed at the higher EY ratio, showed lower yields of about 81%, 77%, and 77%, respectively. The obtained yields of the samples were well consistent with the phenomenon observed during spray-drying in which a greater number of samples E9A1 and E19A1 were adhered to the wall of the spray-drying chamber compared to E1A1. The result could be explained through the lower glass transition temperatures (Tg) of lipids in the EY [32]. The lower transition temperature of the microcapsules according to the higher lipid content in the EY resulted in more extensive adhesion of the microcapsules on the spray-drying chamber and cyclone wall [32,33] and hence lower yields. The production yield of the AA-microcapsules produced in this work is relatively high compared to other reports. Barra et al. (2019) [15] generated AA-microcapsules via spray-drying and using alginate and gum arabic as wall material, the production yield was in the range of ~35–83%. Sartori et al. (2015) [34] encapsulated AA using solid lipids as wall materials, microcapsules were prepared by spray chilling. The production yield was in the range of 62–73%.

Figure 1B shows the encapsulation efficiency (EE) values of microcapsules E19A1, E9A1, E3A1, and E1A1, which were 40%, 40%, 45%, and 64%, respectively. The EE significantly increased when the ratio of EY to AA decreased. This result could be explained by EY containing high lipid content that retarded the solidification of EY to deposit and immobilized the AA. Therefore, a decrease in the EY/AA ratio led to the increase of %EE. The EE of AA-microcapsules obtained in this work was slightly lower than in the other studies. Abbasi et al. (2019) [16] reported that EE of the AA-microcapsules prepared using a composite of gum arabic/maltodextrin/chitosan was varying in the range of 40–80%, depending on the composite ratio. Barra et al. (2019) [15] produced AA microcapsules using alginate and gum arabic as the encapsulation material. The EE was generally as high as 82–98%. In this part, the low EE of the microcapsules could be attributed to the high lipid content of EY, which delay the solidification of the EY-CS encapsulation matrix. Besides, the high lipid content caused high viscosity that adversely affected the EE. An increase in the CS ratio might help enhance the EE of the microcapsules. The effect of the percentage ratio of CS added to coencapsulate the AA with EY was therefore investigated and reported in Section 3.2.

Moist heat resistance of the microcapsules was indicated by the percentage of AA remaining after the microcapsules were directly exposed to moist heat for 5 min. Figure 1C shows the moist heat resistance of the unencapsulated AA and of microcapsules E19A1, E9A1, E3A1, and E1A1, which were 48.7%, 78.8%, 79.0%, 79.0%, and 80.6%, respectively. The result shows that the encapsulation of AA in EY-based microcapsules significantly reduced the loss of AA exposed to the moist heat. The moisture heat resistance of the microcapsules could be due to the fact that EY contained a high content of lipids, which gave the microcapsule a better insulating property against the transfer of high thermal moisture from the environment into the microcapsules [16,35,36]. In addition, the result showed that further increasing the encapsulation ratio of EY to AA from 50/50 did not significantly improve moist heat resistance of AA. In other words, the use of EY at a ratio of 50% of AA was sufficient to achieve a good moist heat protection for the encapsulated AA of 81%. This microencapsulation system exhibited good potential application for other thermal sensitive substance, especially for the protection of probiotics and enzymes in feed application. AA has been chemically improved to have high stability to heat, for instance, after expose of sodium ascorbyl phosphate in 20% aqueous glycerol to 80 °C for 1 day, its retention was 90% [37]. However, the cost of ascorbyl phosphate is high. Furthermore, the shear/friction force and pressure generating during feed production can together with the high moist heat damage the compound. Therefore, the highly stable encapsulated AA with practical cost still serves as a good candidate for aquaculture application. In this study, EY, a natural material with vital biological activities, proved to have good encapsulation properties to protect AA during thermal processes.

Since the sample E1A1 exhibited the highest production yield and encapsulation efficiency with a high moist heat resistance for encapsulated AA, it was selected for the subsequent study to investigate the effects of the ratio of CS to EY on the encapsulation and moist heat resistance of the microcapsules.

### 3.2. Effects of CS as a Coencapsulation Material with EY on Production Yield, Encapsulation Efficiency and Moist Heat Resistance of Microcapsules

**Figure 2:**
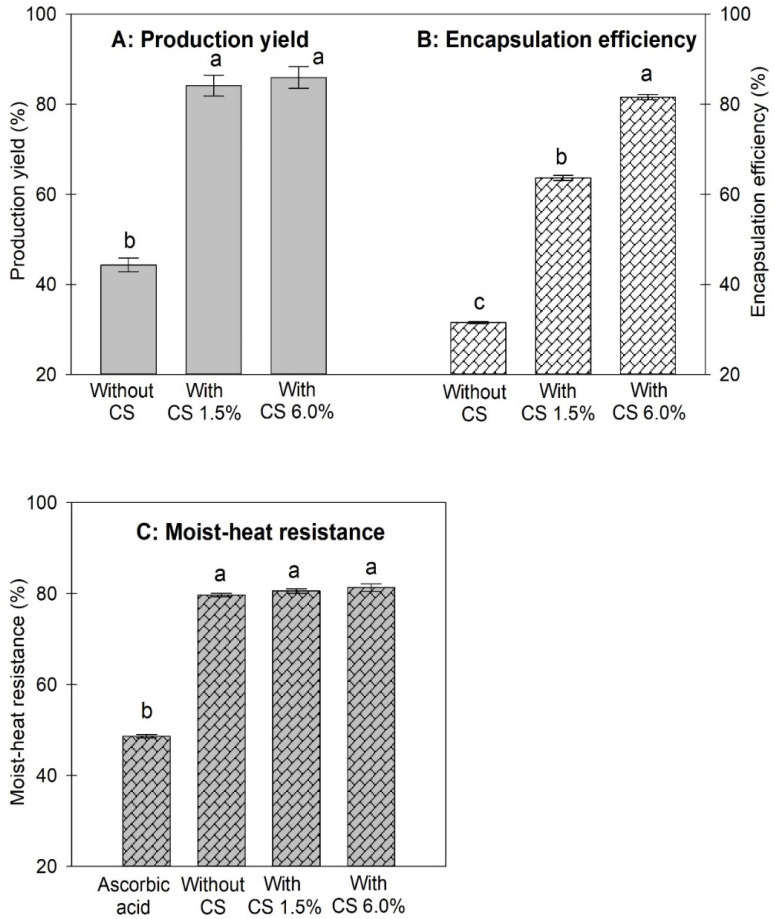
(**A**) Production yield of microcapsules E1A1 produced without or with using CS as a coencapsulation material at a blending ratio of 1.5 or 6% of EY. (**B**) Encapsulation efficiency of microcapsules E1A1 produced without/with using CS as a coencapsulation material at a blending ratio of 1.5 or 6% of EY. (**C**) Moist heat resistance of unencapsulated AA and microencapsulated AA E1A1 produced without or with using CS at a mass ratio of 1.5 or 6% of EY. The different superscripts in each figure indicate significant differences of the values (*p* < 0.05).

In this part, the CS content was studied at 1.5% and 6 % (*w*/*w*) of EY. AA encapsulated in EY without containing CS was used as a control sample. The percentage ratio of EY to AA was set at 50/50 (E1A1) with a total solid content of the spray-drying emulsion of 21.25% (*w*/*w*). The production yields of microcapsules produced with different ratios of CS added as coencapsulating material are shown in Figure 2A. The results show that the use of CS as a coencapsulating material significantly increased the production yield of the microcapsules. When the mass ratio of CS in the microcapsules was increased from 0 to 1.5% and 6.0% of EY, the production yield of the microcapsules increased significantly from 44% to 84% and 86%, respectively. The production yields of the latter two samples (with CS) were relatively high compared to other studies [16,38]. Furthermore, the result was well consistent with the phenomenon observed during spray-drying, the microcapsules produced with CS adhered to the wall chamber much less than that formed without CS. The adhesion of spray-dried particles to the walls of spray-drying chamber and cyclone is generally associated with the glass transition temperature (Tg) [26,32,33,39]. For this result, it might be explained that the positively charged amino groups of CS interacted with the negatively charged counter-ions of the EY molecules (either the lipids or proteins), which lowered the surface charges of the EY, as well as increased Tg of the spray-dried microcapsules. Consequently, a higher production yield of the microcapsules was obtained.

Figure 2B shows the EE of the microcapsules prepared without/with CS. The result shows that EE of the microcapsules was remarkably improved from 32% (without CS) to 64% and 82%, when CS was combined with EY for 1.5% and 6.0%, respectively. The EE of microcapsules produced with 6% CS was relatively high compared to the other studies [15,16]. The result could be explained by the positively charged amine groups of CS interacting with the negatively charged amino groups of EY to promote a denser matrix of the EY-CS composite, thereby resulting in a higher EE. In addition, CS proved to be a good coencapsulating material as it helped to increase the Tg of the spray-dried microcapsules [40], which led to the significant yield improvement.

The effect of CS on the moist heat resistance is shown in Figure 2C. The result shows that the moist heat resistance of the encapsulated AA was significantly improved. However, the combination of CS with EY did not significantly enhance thermal protectability. The study indicated that EY played a key role in moist heat protectability, while CS contributed to the significant improvement of production yield and encapsulation efficiency.

### 3.3. Molecular Characteristics of Microcapsules

**Figure 3:**
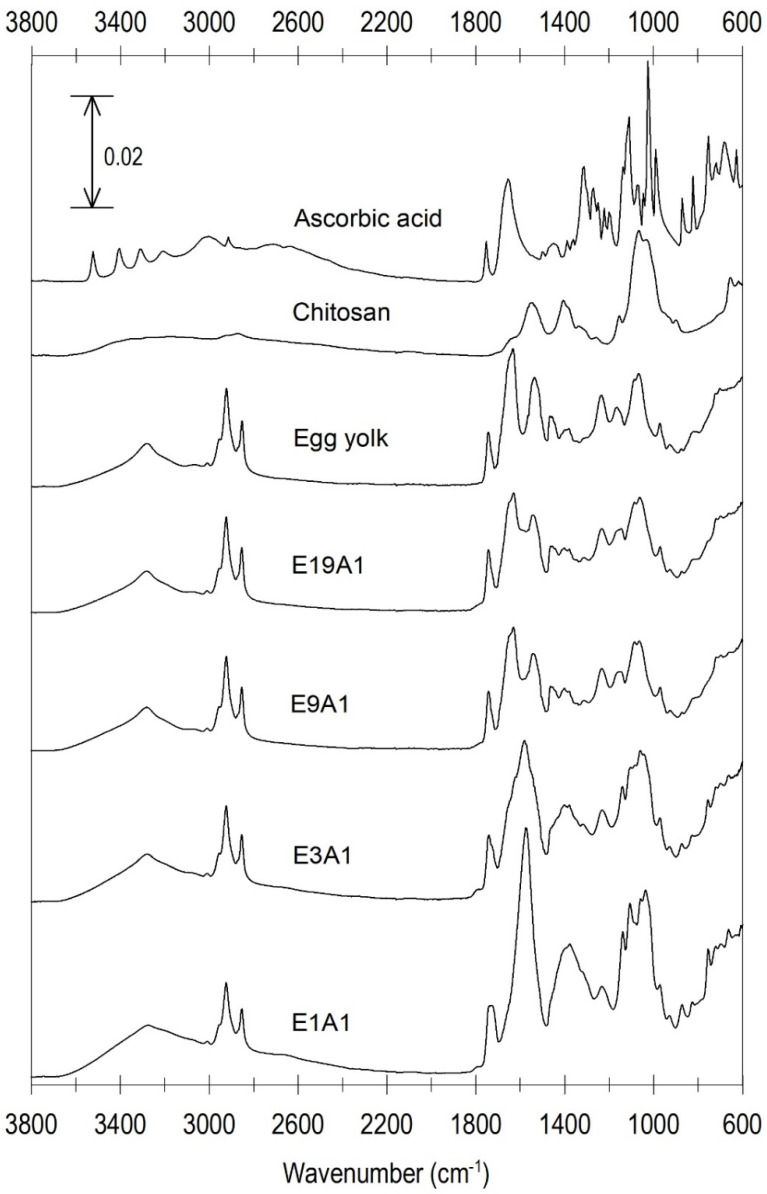
FT–IR spectrograms of ascorbic acid, chitosan, egg yolk and microcapsules E19A1, E9A1, E3A1 and E1A1. The FT–IR spectra were recorded from 3800 to 600 cm^−1^ at a resolution of 4.5 cm^−1^ by 32 scans.

The functional groups of the microcapsule components and their interactions were investigated via FTIR spectroscopy. Figure 3 shows the FTIR spectra of AA, CS, EY and microcapsules E19A1, E9A1, E3A1, and E1A1. The main characteristic peaks of infrared spectrum of EY, detected at 2954–2855, 1745, 1650–1630 and 1535 are associated with the groups -CH and -CH_2_ of the proteins, the carbonyl of the lipid ester, the amide I and the amide II of the secondary protein structure, respectively [41,42,43]. For the band of amide I, the peak at 1652 cm^−1^ indicated the α-helical structure while the band in the range of ~1632–1630 cm^−1^ indicated the intramolecular β-sheet structure. The bands at ~3382 and 3065 cm^−1^ are responsible for the amide A and amide B of EY, respectively. The bands of EY observed at ~1465–1455 and 1405–1380 cm^−1^ are associated with the bending vibrations of CH_2_ and CH_3_ groups of the amino acid side chains, respectively. The peaks at the region of ~1232–820 cm^−1^ with a central peak at ~1064 cm^−1^ are responsible for lecithin; the peaks at 1232, 1165, 1085, 970 and 820 indicated the bonds of P=O, C-O, C-C, N(CH_3_)_3_ and P-O, respectively. For CS, the characteristic peaks of N-acetyl groups were observed at about 1645 cm^−1^ (C=O stretching in amide I), 1553–1550 cm^−1^ (N-H bending in amide II), and ~1335 cm^−1^ (C-N stretching in amide III). The band shown at the region ~1378 cm^−1^ was attributed to the C-H vibration in CH_3_ deformation. The small peak at about 1152 cm^−1^ was associated with the C-O-C bond. The strong bands at ~1065 and 1028 cm^−1^ indicating the saccharide structure were attributed to C-O stretching. In addition, the absorption peaks in the region of ~2923 and 2875 cm^−1^ were associated with the C-H vibration of the glucose rings in polysaccharides [44,45]. The main characteristic peaks of AA were detected at 1751 cm^−1^ (C=O stretching) and 1653 cm^−1^ (C=C ring stretching) [46,47]. Different vibrational bands of AA observed in the region of 1500–1200 cm^−1^ were attributed to the CH_2_ scissoring, twisting, and wagging as well as C-H deformation modes [48]. The band at 1150–1100 cm^−1^ was assigned to C-O-C stretching. The absorption bands at 1020–980 cm^−1^ and 680–640 cm^−1^ were assigned to CH and OH groups, respectively [38].

Due to the high EY ratio in samples E19A1 and E9V, their FTIR spectra pattern are remarkably similar to EY. However, the intensity ratio of the peak at ~1405–1380 cm^−1^ to that at ~1465–1455 was significantly increased. The change was much more evident in samples E3A1 and E1A1. E3A1 exhibited the convoluted characteristic peak at a peak of 1400–1380 with a shoulder at 1465 cm^−1^, the peak (1465–1455 cm^−1^) had disappeared in the sample E1A1. This change suggested the higher amount of amide III structure formation in the samples containing lower ratio of EY. In addition, the spectra showed that two main characteristic peaks of amide I (at 1656 cm^−1^) and amide II (at 1534 cm^−1^) of EY in the sample E3A1 and E1A1 were disappeared, while the new peak at 1573 was evidently exhibited. Moreover, the characteristic peaks of CS at 1551 cm^−1^ and 1337 cm^−1^ were weakly detected in sample E3A1 and were not detected in sample E1A1. On the other hand, the new characteristic peak belonging to the samples E3A1 and E1A1 was detected at 1577 cm^−1^. These results suggested the formation of EY-CS composite in the samples E3A1 and E1A1. Moreover, the FTIR spectra showed that the characteristic peak of lecithin in EY (1232–950 cm^−1^) was more evident when the mass ratio of EY/AA increased. On the other hand, the peak of C=C was weaker and was not detected in the sample E1A1. Overall, the FTIR result suggested the formation of EY-CS-AA composite. The results from FTIR agree well with the previous study of [38]. The authors encapsulated AA in CS matrix and then formed microcapsules via spray-drying process. FTIR indicated the interaction of AA and CS.

### 3.4. Effect of the Ratio of EY to AA on Morphology, Particle Size and Size Distribution of Microcapsules

**Figure 4:**
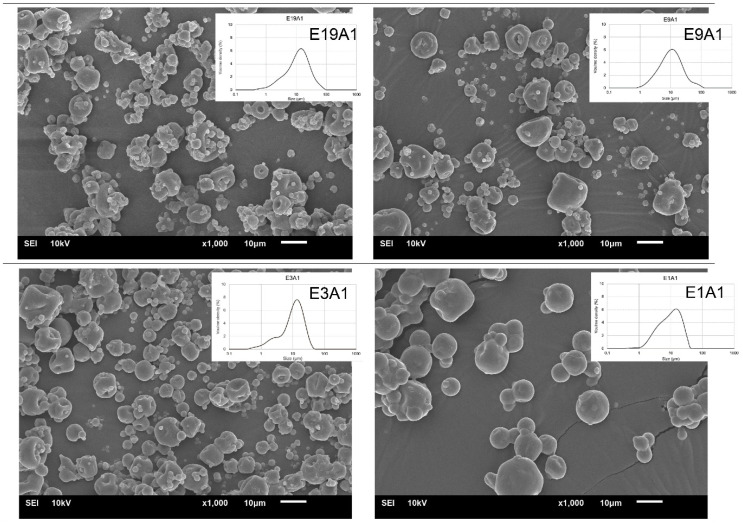
SEM images taken at 1000× magnification of microcapsules E19A1, E9A1, E3A1 and E1A1. All samples were prepared with using CS as a coencapsulation material at a mass ratio of 1.5% of EY.

The morphologies of the microcapsules prepared with different EY/AA ratios are shown in Figure 4. The inset in each microimage presents the particle size and size distribution of microcapsules. The SEM micrographs exhibited that no visible cracks were observed in any samples. However, the higher ratio of EY/AA resulted in structural collapse of the microcapsules. A lower ratio of EY/AA resulted in a smaller dented structure, especially in the case of the sample E1A1; this sample also exhibited very regular shape and smooth surface than the other samples. This might be due to the EY-CS composite formed in the samples E3A1 and E1A1 helped to prevent structural collapse, thereby reducing the surface deformation of the microcapsules. Microcapsules with a smoother surface and less dented structure had higher EE, as expected.

In addition, the results showed that the median particle sizes (D_0.5_) of the microcapsules E19A1, E9A1, E3A1 and E1A1 were 7.7, 7.3, 6.0 and 5.1 µm, respectively. When the mass ratio of EY/AA decreased, the size of microcapsules slightly decreased. Sample E1A1 exhibited the smallest size compared to the other samples. This could be due to the fact that the samples contained lower fat content had a lower viscosity, hence resulted in smaller microcapsules. This result was in consistent with the study of Barra et al. (2019) [15], which showed that smaller microcapsules were obtained from the spray-drying suspension having lower viscosity.

### 3.5. Physical Properties and Stability of Pelleted Feeds in Seawater

#### 3.5.1. Physical Properties of Pelleted Feeds

Water stability of shrimp pellets is an important property because rapid breakage of the pellets in seawater may affect the nutritional values of the diets delivered to the shrimp. The encapsulation matrix mainly consisted of EY, which had a high lipid content that might affect the binding ability of the feed ingredients. Water stability and sinking velocity of the pellets fortified with the microcapsules (E1A1) were thus evaluated.

Table 2 presents the results on physical properties of feed samples, i.e., moisture content, bulk density and sinking velocity, of the plain pelleted feed, the pelleted feed containing unencapsulated AA, the pelleted feed containing encapsulated AA, and the commercial feed pellets. The addition of encapsulated AA to the shrimp feed did not affect the moisture content, sinking velocity or density of the resulting pelleted feed. The physical properties of the samples were slightly lower than those of selected commercial feed pellets according to the limit of the extrusion used to prepare the pelleted feed; however, all properties of the samples were in accordance with the regulation of Thai Industrial Standards Institute (TISI) for dry pelleted feed products.

#### 3.5.2. Stability of Pelleted Feeds in Seawater

**Figure 5:**
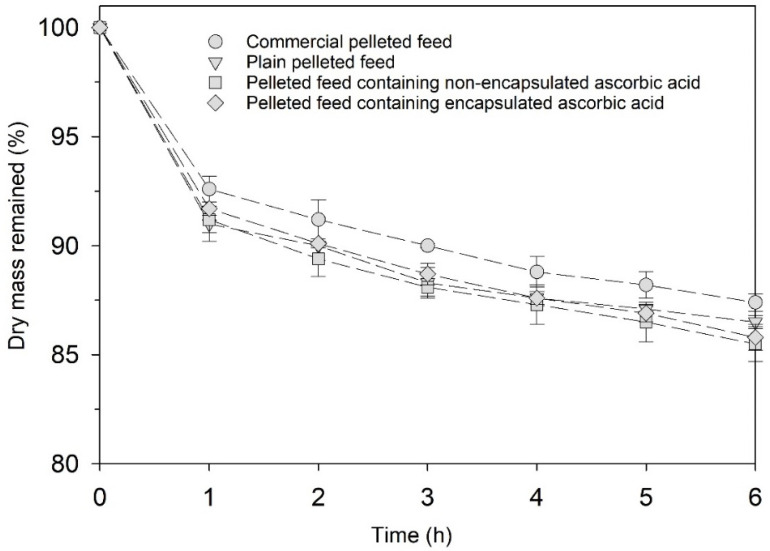
Stability of the pelleted shrimp feeds in seawater (plain pelleted feed, pelleted feed containing unencapsulated AA and pelleted feed containing microencapsulated AA). The stability was reported in terms of the mass ratio of the pelleted feed remained after exposure to seawater to its initial mass.

Figure 5 shows the stability of pelleted shrimp feeds in the seawater. The results showed that the stability in seawater for 6 h was in the range of 85–87% for all the pelleted feed samples, which met the TISI standards for pelleted shrimp feed. The fortified microcapsules had no adverse effect on the stability of the pelleted feeds in the seawater. Although the EY ratio in the feed was only 0.24% (d.b.), EY contains high lipid content. Therefore, shelf life in terms of rancidity of the feed should be further investigated. Besides, a possible impact of the microcapsule-fortified feed to compromise the seawater quality was noted to evaluate in the future study.

#### 3.5.3. Losses of Fortified AA during the Production Process of Feed Pellets and in Seawater

Table 3 shows the retention of fortified AA during the production of pelleted feed and by leaching into seawater. The amounts of AA remaining in the pellets fortified with unencapsulated and encapsulated AA after extrusion were 33.7% and 85.8%, respectively. After drying, the percentage in the former and latter samples decreased to 17.4% and 66.6%, respectively. The results also showed that the final amount of AA remaining in the shrimp feed after extrusion, hot air drying and 6 h exposure to seawater was 3% for unencapsulated AA-fortified feed and 47% for encapsulated AA-fortified feed. The study evidently indicated that encapsulation of AA in EY-CS composite matrix significantly minimizes losses of AA during in feed processing and 6 h exposure to seawater.

## 4. Conclusions

This study for the first time reports the use of EY in combination with CS as an encapsulation material to effectively protect the thermal dissociation of bioactive compound during feed processing. In addition, the encapsulation system was feasible for use in aquafeed application to minimize leaching of the encapsulated compounds in seawater. In the study, EY proved to be a key component on thermal protection of the encapsulated compound. CS in a small ratio of only 1.5% of EY was needed as a coencapsulation material to improve the morphology, production yield, and encapsulation efficiency of the microcapsules. The production yield and EE of the microcapsules developed in this study were relatively high compared to other works. Moist heat resistance of the encapsulated AA was up to 82%. In addition, the encapsulated AA could be fortified in feed pellets without adverse impacts on bulk density, water stability and sinking velocity of the shrimp feed. Apart from the good thermal protectability, the microcapsules were mainly composed of EY that has biofunctions vital to aquatic animals. The microcapsule thus possesses high potential application of highly nutritious shrimp feed production. The digestion and biological activity in shrimp of the encapsulated AA-fortified feed is being investigated in our lab. 

## Figures and Tables

**Table 1:** Composition of the shrimp feed samples (dry basis).

Ingredients	Composition of Shrimp Feed Sample (g/100 g Feed)		
	Plain Pelleted Feed	Pelleted Feed Containing Unencapsulated AA	Pelleted Feed Containing Encapsulated AA
Cereal	40.00	39.92	39.80
Soybean meal	30.00	29.94	29.85
Fishmeal	12.00	11.98	11.94
Shrimp head meal	10.00	9.98	9.95
Fish oil	4.00	3.98	3.98
Dicalcium phosphate	2.00	2.00	1.99
Corn starch	2.00	2.00	1.99
AA	0	0.20	0
Microcapsule E1A1 (AA payload in microcapsules = 40%)	0	0	0.50 *
Total	100.00	100.00	100.00

* The percentage mass fraction of EA, CS and AA in the feed were 0.24, 0.02 and 0.24, respectively.

**Table 2:** Physical properties of pelleted feed samples.

Physical Properties	Plain Pelleted Feed	Pelleted Feed Containing Unencapsulated AA	Pelleted Feed Containing Encapsulated AA	Commercial Pelleted Feed
Moisture content (d.b., %)	11.03 ± 0.67 ^a^	10.98 ± 0.41 ^a^	11.21 ± 0.33 ^a^	11.28 ± 0.42 ^a^
Bulk density (g/cm^3^)	0.52 ± 0.01 ^b^	0.56 ± 0.02 ^b^	0.53 ± 0.02 ^b^	0.63 ± 0.01 ^a^
Sinking velocity (cm/s)	4.21 ± 0.78 ^b^	4.49 ± 0.42 ^b^	4.31 ± 0.46 ^b^	5.01 ± 0.38 ^a^

^a,b^ Values with different letters in the same row were significantly different (*p* < 0.05).

**Table 3:** Retention of AA in pelleted feed at each step of feed processing and during 6-h of the exposure to seawater.

Process	Retention of AA in Pelleted Feed (%)	
	Unencapsulated AA	Encapsulated AA
Before extrusion	100.0	100.0
After extrusion	33.7	85.8
After drying at 90 °C for 1 h	17.4	66.6
After exposure to seawater for 1 h	12.3	63.5
After exposure to seawater for 3 h	8.1	57.6
After exposure to seawater for 6 h	3.0	46.9
